# Reconstruction of the Diaminopimelic Acid Pathway to Promote L-lysine Production in *Corynebacterium glutamicum*

**DOI:** 10.3390/ijms22169065

**Published:** 2021-08-23

**Authors:** Ning Liu, Ting-Ting Zhang, Zhi-Ming Rao, Wei-Guo Zhang, Jian-Zhong Xu

**Affiliations:** 1 The Key Laboratory of Industrial Biotechnology, Ministry of Education, School of Biotechnology, Jiangnan University, Wuxi 214122, China; 6190206010@stu.jiangnan.edu.cn (N.L.); ln576678072@163.com (T.-T.Z.); zhangwg@jiangnan.edu.cn (W.-G.Z.); 2 National Engineering Laboratory for Cereal Fermentation Technology (NELCF), Jiangnan University, 1800# Lihu Road, Wuxi 214122, China

**Keywords:** L-lysine biosynthesis, *Corynebacterium glutamicum*, diaminopimelic acid pathway, diaminopimelate dehydrogenase, ammonium supply

## Abstract

The dehydrogenase pathway and the succinylase pathway are involved in the synthesis of L-lysine in *Corynebacterium glutamicum*. Despite the low contribution rate to L-lysine production, the dehydrogenase pathway is favorable for its simple steps and potential to increase the production of L-lysine. The effect of ammonium (NH_4_^+^) concentration on L-lysine biosynthesis was investigated, and the results indicated that the biosynthesis of L-lysine can be promoted in a high NH_4_^+^ environment. In order to reduce the requirement of NH_4_^+^, the nitrogen source regulatory protein AmtR was knocked out, resulting in an 8.5% increase in L-lysine production (i.e., 52.3 ± 4.31 g/L). Subsequently, the dehydrogenase pathway was upregulated by blocking or weakening the tetrahydrodipicolinate succinylase (DapD)-coding gene *dapD* and overexpressing the *ddh* gene to further enhance L-lysine biosynthesis. The final strain XQ-5-W4 could produce 189 ± 8.7 g/L L-lysine with the maximum specific rate (*q*_Lys,max._) of 0.35 ± 0.05 g/(g·h) in a 5-L jar fermenter. The L-lysine titer and *q*_Lys,max_ achieved in this study is about 25.2% and 59.1% higher than that of the original strain without enhancement of dehydrogenase pathway, respectively. The results indicated that the dehydrogenase pathway could serve as a breakthrough point to reconstruct the diaminopimelic acid (DAP) pathway and promote L-lysine production.

## 1. Introduction

L-lysine is an essential amino acid widely used in food, animal feed, medicine, cosmetics, and other industries [1]. Methods to produce L-lysine include albuminolysis, chemical method, enzymic method, and microbial fermentation. Microbial fermentation uses renewable feedstock and produces low amounts of pollutants [2]. Therefore, L-lysine is mainly produced by microbial fermentation in the industries, with *C. glutamicum* and *Escherichia coli* being the most commonly used strains [1].

**Figure 1:**
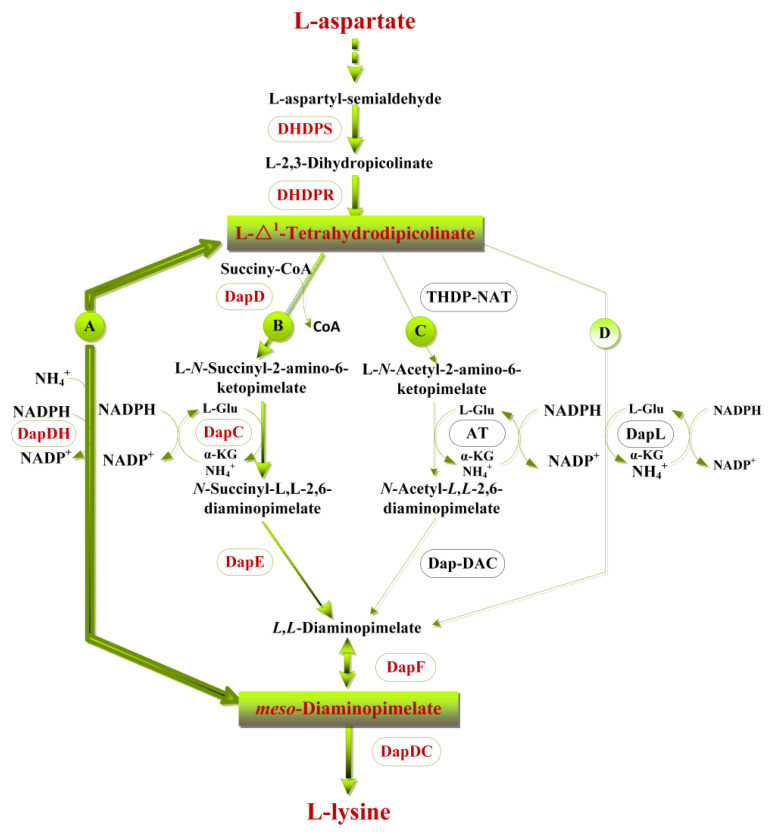
The DAP pathway of L-lysine synthesis. (**A**) Dehydrogenase pathway, (**B**) succinylase pathway, (**C**) acetylase pathway, (**D**) aminotransferase pathway. Abbreviations: DHDPS Dihydrodipicolinate synthetase, DHDPR Dihydrodipicolinate reductase, DapDH Diaminopimelate dehydrogenase, DapD Tetrahydrodipicolinate *N*-succinyltransferase, DapC Succinyl-amino-ketopimelate transaminase, DapE *N*-succinyl-diaminopimelate desuccinylase, DapF Diaminopimelate epimerase, THDP-NAT Tetrahydrodipicolinate acetylase, AT *N*-acetylaminoketopimelate aminotransferase, NAD-DAC *N*-acetyl-diaminopimelate deacetylase, DapL Tetrahydrodipicolinate aminotransferase.

The L-lysine biosynthesis includes two pathways, the diaminopimelic acid (DAP) pathway and the α-aminoadipic acid (AAA) pathway. In the AAA pathway, L-lysine is synthesized from α-ketoglutarate and acetylcoenzyme A (acetyl-CoA) in which α-aminoadipic acid serves as an intermediate metabolite. The AAA pathway is commonly found in yeast, fungi, and some species in the domain Archaea [3,4]. In the DAP pathway, however, L-lysine is synthesized from aspartate and pyruvate in which *meso*-diaminopimelic acid (*meso*-DAP) serves as an intermediate metabolite (Figure 1) [5]. Commonly found in archaea, algae, fungi, plants, and bacteria [6], the DAP pathway starts with the biosynthesis of L-Δ^1^-tetrahydrodipicolinate (THDPA) from L-aspartate, which is then converted into *meso*-DAP, and finally, L-lysine is produced with diaminopimelate deacetylase (DAPDC, EC:4.1.1.20) as the catalyst [7]. The conversion into *meso*-DAP is the essential step that distinguishes the four DAP pathway variations, the succinylase pathway, acetylase pathway, dehydrogenase pathway, and aminotransferase pathway [8]. The four variants share the common steps of converting L-aspartate to THDPA by aspartokinase (AK, EC:2.7.2.4), aspartic semialdehyde dehydrogenase (AsaDH, EC:1.2.1.11), dihydrodipicolinate synthetase (DHDPS, EC:4.3.3.7), and dihydrodipicolinate reductase (DHDPR, EC:1.17.1.8) in turn. Different enzymes are involved in the four different variant pathways to produce *meso*-DAP (Figure 1). The succinylase pathway is the most common in most bacteria including *E. coli* [9]. The acetylase pathway is found only in some *Bacillus*, and the dehydrogenase pathway only exists in some Gram-positive bacteria (i.e., *Corynebacterium* and *Bacillus*) [10] and plants (i.e., *Glycine* and *Zea*) [11]. The aminotransferase pathway is found in *Cyanobacteria*, *Chlamydia*, *Methanothermobacter thermautotrophicus,* and *Arabidopsis thaliana* [12,13,14]. Among the four variants, THDPA is directly converted into *meso*-DAP in the dehydrogenase pathway. Therefore, the dehydrogenase pathway is more favorable in situations where energy is limited [15]. However, L-lysine accumulation in the dehydrogenase pathway requires high NH_4_^+^ concentrations [16] due to the low affinity of diaminopimelate dehydrogenase (DapDH, E.C. 1.4.1.16) for the substrate. Thus, the application of the dehydrogenase pathway is limited [15].

*C. glutamicum* is a Gram-positive bacterium isolated from soil in 1957 [17,18]. *C. glutamicum* is often used to produce amino acids commercially, such as L-glutamic acid, L-lysine and L-arginine [19]. Interestingly, two variants of the DAP pathway are found in *C. glutamicum*, the succinylase pathway, and the dehydrogenase pathway. According to Figure 1, *meso*-DAP is biosynthesized from THDPA in one step via the dehydrogenase pathway [20] and in four steps via the succinylase pathway, both of which involve NH_4_^+^. In addition, the L-lysine biosynthesis efficiency of both pathways depends on the concentration of NH_4_^+^ in the medium [16]. L-lysine was produced mainly through the dehydrogenase pathway at first but then entirely through the succinylase pathway [16] as the concentration of NH_4_^+^ decreases during the fermentation, resulting in the decrease in the dehydrogenase activity [16,21]. Sonntag et al. reported that 33% of L-lysine was synthesized via the dehydrogenase pathway and 66% via the succinylase pathway in *C. glutamicum* [22]. Although the succinylase pathway is important for increasing the titer of L-lysine, the dehydrogenase pathway has potential in improving the production intensity of L-lysine as only one step is required to biosynthesize *meso*-DAP. Hence the question: does upregulating the dehydrogenase pathway promote L-lysine production in *C. glutamicum*?

In this study, the *C. glutamicum* XQ-5 strain was developed, which took the dehydrogenase pathway for *meso*-DAP biosynthesis to promote L-lysine production. The DAP pathway of *C. glutamicum* XQ-5 was reconstructed to upregulate the dehydrogenase pathway in L-lysine biosynthesis during the fermentation process. The strategies of this study include as follows: (1) investigating the effect of different NH_4_^+^ concentrations on L-lysine production; (2) alleviating the nitrogen limitation to improve the efficiency of L-lysine production; (3) rationally regulating the two pathways to promote L-lysine production. As a result, a recombinant strain *C. glutamicum* XQ-5-W4 (i.e., *C. glutamicum* XQ-5-*dapD*^W^Δ*amtR*/pEC-*ddh*) derived from *C. glutamicum* XQ-5 was obtained, which produced 189 ± 8.7 g/L L-lysine with the maximum specific rate (*q*_Lys,max._) of 0.35 ± 0.05 g/(g·h) in a 5-L jar fermenter, which were 25.2% and 59.1% higher than that of the original strain *C. glutamicum* XQ-5, respectively.

## 2. Results and Discussion

### 2.1. The Effects of Different Ammonium (NH_4_^+^) Concentrations on L-lysine Production

**Figure 2:**
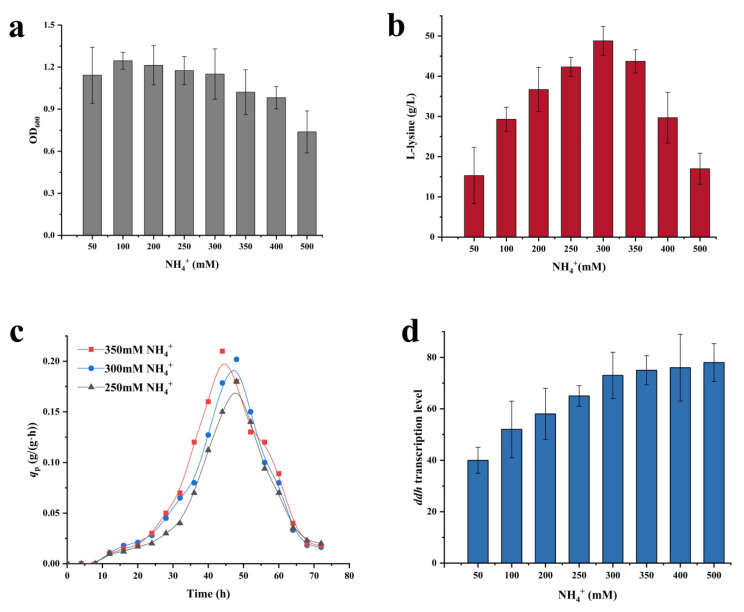
The effects of different ammonium (NH_4_^+^) concentrations on L-lysine synthesis. (**a**) Cell growth (OD_600_) of strain XQ-5 at different NH_4_^+^ concentrations. The cell concentration after 25 fold dilution was measured at OD_600._ (**b**) L-lysine production of strain XQ-5 at different NH_4_^+^ concentrations. (**c**) The *q*_Lys_ of strain XQ-5 at different NH_4_^+^ concentrations. (**d**) Transcription level of gene *ddh* at different NH_4_^+^ concentrations. All data represent values of three determinations of triplicate independent experiments.

Nitrogen is one of the essential nutrients for living cells. The nitrogen utilization is very important for the growth of bacteria. Ammonium (NH_4_^+^) is the standard component of the growth medium and the preferred nitrogen source for many bacteria. Both variants of the DAP pathway in *C. glutamicum* require the participation of NH_4_^+^. Therefore, the effect of NH_4_^+^ on L-lysine production was investigated. To do this, eight NH_4_^+^ solutions of different concentrations (i.e., 50, 100, 200, 250, 300, 350, 400, 500 mM) were added to the culture mediums and the cell growth, L-lysine production as well as *q*_Lys_ were monitored. The results showed that the growth of strain XQ-5 was significantly inhibited from high NH_4_^+^ concentration, but the effect of low NH_4_^+^ concentration is relatively insignificant (Figure 2a). Xu et al. observed similar results in which high NH_4_^+^ concentration was toxic to the bacteria [23]. Interestingly, too high or too low NH_4_^+^ concentration is not conducive to L-lysine accumulation (Figure 2b). The L-lysine yield was relatively high at NH_4_^+^ concentrations of 250 mM, 300 mM and 350 mM, which were 42.3 ± 2.31 g/L, 48.2 ± 3.54 g/L, and 43.9 ± 3.78 g/L, respectively. Interestingly, the maximum specific rate (*q*_Lys,max._) was found with a 350 mM NH_4_^+^ solution (i.e., 0.22 ± 0.03 g/(g·h)), which was 10% higher than that with a 300 mM NH_4_^+^ solution (i.e., 0.20 ± 0.01 g/(g·h)) (Figure 2c). These results indicated that an appropriate increase in NH_4_^+^ supply is necessary for the effective biosynthesis of L-lysine. Previous research reported that the dehydrogenase pathway is only active at high NH_4_^+^ concentration [22]. Therefore, increasing the concentration of NH_4_^+^ may upregulate the dehydrogenase pathway and promote L-lysine biosynthesis. The transcription level of the DapDH-coding gene *ddh* was measured. As might be expected, the transcription level of *ddh* gene increased with the increase of NH_4_^+^ concentration within a certain range (Figure 2d). It is safe to conclude that the increase of *q*_Lys,max_ at high NH_4_^+^ concentration was related to the increase in *ddh* gene transcription level, and upregulating the dehydrogenase pathway is conducive to promoting L-lysine biosynthesis.

### 2.2. Effect of The Upregulated Dehydrogenase Pathway on L-lysine Biosynthesis

**Figure 3:**
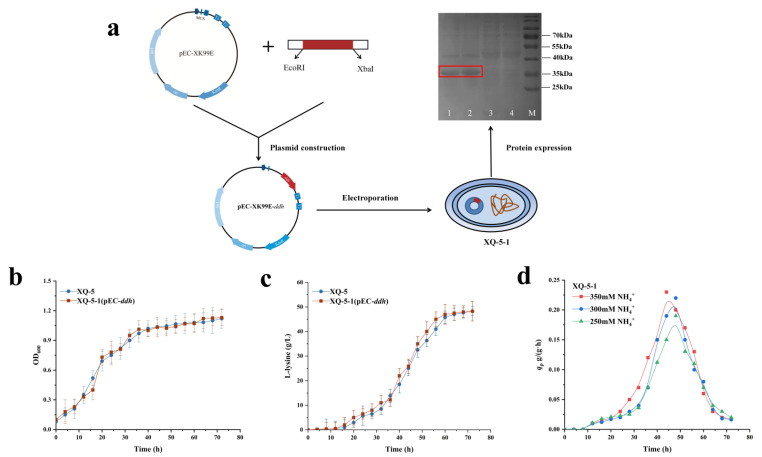
Effect of dehydrogenase pathway on L-lysine synthesis. (**a**) The construction process of strain XQ-5-1 and SDS-PAGE analysis of DapDH (i.e., DapDH: 35 kDa). Lane M, protein ruler; lane 1,2: crude enzyme extract (i.e., strain XQ-5-1); lane 3,4: crude enzyme extract (i.e., strain XQ-5). (**b**) Cell growth (OD_600_) of strain XQ-5 and XQ-5-1 in shake flask culture. The cell concentration after a 25-fold dilution was measured at OD_600_. (**c**) L-lysine production of strain XQ-5 and XQ-5-1 in shake flask culture. (**d**) The *q*_Lys_ of strain XQ-5-1 in shake flask culture. The data represent mean values and standard deviations obtained from three independent cultivations.

As mentioned above, upregulating *ddh* gene promotes the biosynthesis of L-lysine. It is speculated that upregulating the dehydrogenase pathway may further increase L-lysine production. In order to enhance the effect of the dehydrogenase pathway on L-lysine production, the *ddh* gene was overexpressed to investigate whether the *q*_Lys_ and L-lysine production were improved. The *ddh* gene was ligated into the plasmid pEC-XK99E, which was then introduced into strain XQ-5 to give the target strain XQ-5/pEC-*ddh* (i.e., strain XQ-5-1) (Figure 3a). The growth rate, L-lysine production and *q*_Lys_ of strain XQ-5-1 were measured at shake flask fermentation for 72 h, and the effect of the upregulated dehydrogenase pathway on L-lysine production was investigated.

According to Figure 3b, the growth of strain XQ-5-1 was not affected, remaining almost the same as the original strain. In addition, the final L-lysine production of strain XQ-5-1 was 49.3 ± 3.21 g/L, slightly increased compared with strain XQ-5 (i.e., 48.2 ± 3.54 g/L) (Figure 3c). The results showed that the upregulated dehydrogenase pathway had little effect on L-lysine production. Similar results were also found in previous reports in which the overexpression of *ddh* gene on plasmid had no effect on the L-lysine production of *C. glutamicum* [24]. It is speculated that the intracellular NH_4_^+^ concentration limits the increase of L-lysine production in strains with overexpressed *ddh*. Due to the low affinity to NH_4_^+^, the dehydrogenase pathway is only adoptable at high NH_4_^+^ concentration [16]. However, it is heartening that XQ-5-1 produced the same amount of L-lysine as the original strain in a shorter time (Figure 3c). With the same NH_4_^+^ concentration, the *q*_Lys,max_ of XQ-5-1 was 5–10% higher than that of the original strain (Figure 2c and Figure 3d). Previous research suggested that the level of extracellular NH_4_^+^ concentration is higher than the intracellular NH_4_^+^ concentration as the utilization of nitrogen is controlled by AmtR [25]. Then, is the increase in intracellular NH_4_^+^ concentration beneficial to L-lysine production?

### 2.3. Effect of Gene amtR Deletion on NH_4_^+^ Utilization and L-lysine Synthesis

**Figure 4:**
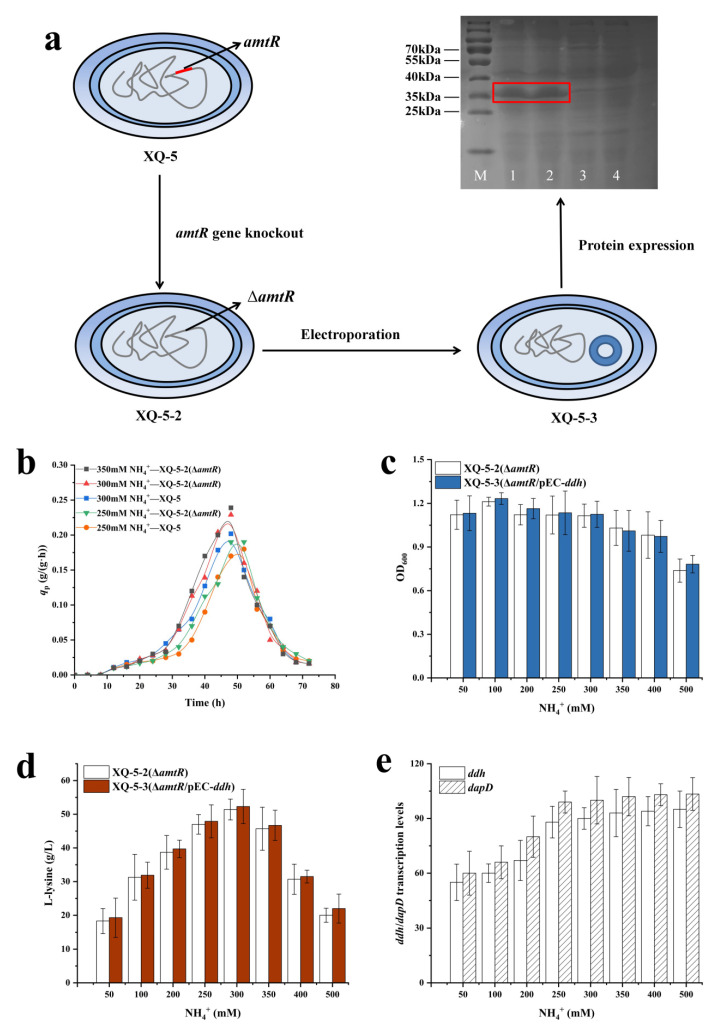
Effect of alleviating nitrogen source restriction on L-lysine synthesis. (**a**) The construction process of strain XQ-5-3 and SDS-PAGE analysis of DapDH (i.e., 35 kDa). Lane M, protein ruler; lane 1,2: crude enzyme extract (i.e., strain XQ-5-3); lane 3,4: crude enzyme extract (i.e., strain XQ-5). (**b**) The *q*_Lys_ of strain XQ-5 and strain XQ-5-1 under different NH_4_^+^ concentrations. (**c**) Cell growth (OD_600_) of strain XQ-5-1 and XQ-5-2 in shake flask culture. The cell concentration after 25 fold dilution was measured at OD_600_. (**d**) L-lysine production of strain XQ-5-1 and XQ-5-2 in shake flask culture. (**e**) Transcription levels of *ddh* and *dapD* in strain XQ-5-3. All data represent values of three determinations of triplicate independent experiments.

As mentioned above, L-lysine production showed no significant increase with the overexpression of *ddh*, possibly due to the low intracellular NH_4_^+^ concentration. To address this limitation, the regulatory proteins controlling the utilization of NH_4_^+^ should be inactivated. In *C. glutamicum*, the expression of genes responding to nitrogen utilization is controlled by the TetR-type regulator AmtR [25]. In contrast with most TetR-type regulators, the dissociation of AmtR from its target promoters is triggered by complex formation of the PII-type signal transduction protein GlnK rather than by the binding of a low-molecular mass ligand [26]. The transcriptional regulator AmtR responses to changes in nitrogen levels, thus at least 35 genes involved in nitrogen utilization and metabolism needed to be regulated [27]. In order to increase the concentration of NH_4_^+^, the AmtR-coding gene *amtR* was deleted to give the target strain *C. glutamicum* XQ-5-Δ*amtR* (i.e., XQ-5-2) (Figure 4a). Interestingly, the *q*_Lys,max_ of strain XQ-5-2 at 250 mM NH_4_^+^ (i.e., 0.19 ± 0.02 g/(g·h)) was similar as the strain XQ-5 at 300 mM NH_4_^+^ (i.e., 0.20 ± 0.01 g/(g·h)). Moreover, the *q*_Lys,max_ of strain XQ-5-2 was almost the same at 300 mM (i.e., 0.22 ± 0.03 g/(g·h)) and 350 mM NH_4_^+^ (i.e., 0.23 ± 0.04 g/(g·h)) (Figure 4b). In addition, the highest L-lysine production (i.e., 52.3 ± 4.31 g/L) of strain XQ-5-2 was obtained at 300 mM NH_4_^+^, which was 8.5% higher than that of strain XQ-5 (i.e., 48.2 ± 3.54 g/L) (Figure 4d). Previous results also indicated that the deletion of the *amtR* gene increased the yield of L-lysine [28]. These results indicated that the alleviation of nitrogen restriction increased the intracellular NH_4_^+^ concentration.

Subsequently, the plasmid pEC-XK99E*-ddh* was introduced into strain XQ-5-2 to give the target strain XQ-5-2/pEC-*ddh* (i.e., strain XQ-5-3). As expected, the growth of strain XQ-5-2 was inhibited from high NH_4_^+^ concentration (Figure 4c). It should be noted that the L-lysine yield of strain XQ-5-3 was 53.8 ± 3.98 g/L, which is similar to that of strain XQ-5-2 (i.e., 52.3 ± 4.31 g/L) (Figure 4d). These results indicated that the overexpression of *ddh* gene did not significantly increase the L-lysine production of strain XQ-5-2, possibly due to the fact that the dehydrogenase pathway is still not the dominant pathway for L-lysine production of strain XQ-5-3. According to Figure 1, the dehydrogenase pathway is a reversible reaction. In addition, previous reports pointed out that DapDH is highly specific to *meso*-DAP, while *L*,*L*-DAP and *D*,*D*-DAP are competitive inhibitors [29]. Theoretically, *L*,*L*-DAP, an intermediate in the succinylase pathway, inhibits the reverse reaction of DapDH [29]. Thus, the succinylase pathway was upregulated to supply *L*,*L*-DAP during the overexpression of *ddh*. In order to confirm this conjecture, the transcription levels of *ddh* and *dapD* were measured in strain XQ-5-3. The results showed that the transcription levels of *ddh* and *dapD* increased with the increase of NH_4_^+^ concentration within a certain range (Figure 4e). The transcription level of *ddh* was 82% higher at high NH_4_^+^ concentration (i.e., 500 mM) than that at low NH_4_^+^ concentration (i.e., 50 mM). Similarly, the transcription level of *dapD* was 92% higher at high NH_4_^+^ concentration (i.e., 500 mM) than that at low NH_4_^+^ concentration (i.e., 50 mM). Moreover, it is reported that the expression level of *dapD* gene increases after the deletion of *amtR* gene [28]. These results indicated that the upregulated *dapD* expression level comes with the upregulated *ddh* expression level. However, DapD (tetrahydrodipicolinate succinylase, E.C. 2.3.1.117) has a high affinity with the substrate THDPA [15], making the succinylase pathway the main pathway for biosynthesizing L-lysine rather than the dehydrogenase pathway in spite of the overexpression of *ddh*.

### 2.4. Blocking the Succinylase Pathway to Upregulate the Dehydrogenase Pathway

**Figure 5:**
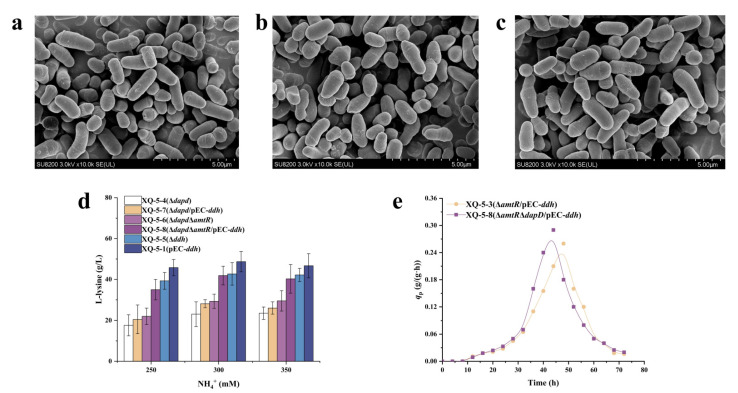
Blocking succinylase pathway to redirection of flux in dehydrogenase pathway. (**a**) FESEM of strain XQ-5. (**b**) FESEM of strain XQ-5-4. (**c**) FESEM of strain XQ-5-5. (**d**) L-lysine production of strain XQ-5-1, XQ-5-4, XQ-5-5, XQ-5-6, XQ-5-7, XQ-5-8 in shake flask culture. (**e**) The q_Lys_ of strain XQ-5-3 and strain XQ-5-8 at 300 mM NH_4_^+^ concentration. All data represent values of three determinations of triplicate independent experiments.

Based on the above results, increasing the expression of *ddh* gene in the dehydrogenase pathway showed no significant effect on L-lysine production, possibly due to the fact that the expression level of *dapD* is increased with the overexpression of *ddh*. In order to upregulate the dehydrogenase pathway, the succinylase pathway was blocked. The succinylase pathway involves enzymes such as DapC (succinyl-amino-ketopimelate-coding gene *dapC*), DapE (*N*-succinyl-diaminopimelate desuccinylase-coding gene *dapE*), and DapF (diaminopimelate epimerase-coding gene *dapF)*, respectively [30,31,32]. Previous research indicated that the genes *dapC* and *dapE* are dispensable for L-lysine overproduction in shake-flask cultures [33,34], whereas the genes *dapF* and *dapD* are indispensable for the succinylase pathway [15,33]. DapD is the first key enzyme in the succinylase pathway, thus DapD was inactivated to block the succinylase pathway, hence the DapD-deficient strain XQ-5-Δ*dapD* (i.e., strain XQ-5-4). As a control, the DapDH-deficient strain XQ-5-Δ*ddh* (i.e., strain XQ-5-5) was also constructed. The cell morphology of the three strains (i.e., a:strain XQ-5, b:strain XQ-5-4, c:strain XQ-5-5) were observed with FESEM, and the results indicated almost no change in cell morphology compared to the original strain (Figure 5a–c). It is well-known that *meso*-DAP connects the glycan backbone on the cell wall of many bacteria to give them shape and rigid structure [8]. These results showed that *meso*-DAP synthesized by either of these two pathways had met the needs of cell structure.

In order to investigate the L-lysine production of different strains under the different NH_4_^+^ concentrations, three NH_4_^+^ solutions of different concentrations (i.e., 250 mM, 300 mM, and 350 mM) were used in the test. According to Figure 5d, L-lysine production of strain XQ-5-4 decreased significantly, especially at low NH_4_^+^ concentration (i.e., 17.6 ± 5.11 g/L), indicating that *dapD* gene is essential for the succinylase pathway and L-lysine production. By contrast, the L-lysine production of strain XQ-5-5 (i.e., 39.3 ± 4.11 g/L) decreased slightly. These results have once again proven that the succinylase pathway is the main pathway for L-lysine production rather than the dehydrogenase pathway [22]. It should be noted that the L-lysine production of strain XQ-5-4 increased at high NH_4_^+^ concentration (Figure 5d), possibly due to the fact that the one-step dehydrogenase pathway compensated the L-lysine production at high NH_4_^+^ concentration when the succinylase pathway was downregulated [34]. Among the recombinant strains, strain XQ-5-Δ*dapD*Δ*amtR*/pEC-*ddh* (i.e., strain XQ-5-8) accumulated the highest L-lysine production (i.e., 41.9 ± 4.57 g/L) if not counting the strains with succinylase pathway (Figure 5d). It is worth noting that the overexpression of *ddh* gene in strain XQ-5-6 increased the L-lysine production (300 mM) from 29.3 ± 3.49 g/L (strain XQ-5-6) to 41.9 ± 4.57 g/L (strain XQ-5-8). Interestingly, although the L-lysine yield of strain XQ-5-8 (i.e., 41.9 ± 4.57 g/L) was lower than that of strain XQ-5-3 (i.e., 53.8 ± 3.98 g/L), the *q*_Lys,max._ of strain XQ-5-8 (i.e., 0.30 ± 0.04 g/(g·h)) was 20% higher than that of strain XQ-5-3 (i.e., 0.25 ± 0.03 g/(g·h)) (Figure 5e), possibly due to the fact that the L-lysine precursor (i.e., *meso*-DAP) was biosynthesized in one step rather than four steps [35] since the dehydrogenase pathway is the only pathway for L-lysine production of strain XQ-5-8. These results also showed that the dehydrogenase pathway has potential to increase L-lysine production. Taken together, these results indicated that blocking the succinylase pathway is beneficial to upregulating the dehydrogenase pathway, thus improving the *q*_Lys,max_. However, the L-lysine production in strains with blocked the succinylase pathway decreased. Two reasons were proposed: (1) More NH_4_^+^ were required to produce L-lysine in the dehydrogenase pathway. Previous researches indicated that *C. glutamicum* strain with the dehydrogenase pathway alone cannot produce L-lysine at low NH_4_^+^ concentration [36] due to the low affinity of the dehydrogenase pathway to NH_4_^+^ in the substrate [16]; (2) the dehydrogenase pathway is a reversible process. *L*,*L*-DAP cannot be synthesized while blocking the succinylase pathway, thus relieving the competitive inhibition of the reverse reaction [29]. These proposed reasons have been validated by examining the concentration of the by-products (Table 1). As might be expected, strain XQ-5-8 had the highest by-product concentration at high NH_4_^+^ concentration (Table 1). All of the above mentioned results indicated that blocking the succinylase pathway is beneficial to improve the production intensify of L-lysine, but unhelpful for increasing the yield of L-lysine.

### 2.5. Weakening the dapD Gene Makes the Metabolic Flux of the Two Pathways Reach the Best Balance

As mentioned above, L-lysine production decreased significantly while blocking the succinylase pathway (Figure 5d) and the amount of by-products increased because of the lack of L,L-DAP (Table 1). To address this problem, the key enzyme DapD in the succinylase pathway was weakened to balance the flux between the succinylase pathway and the dehydrogenase pathway.

**Figure 6:**
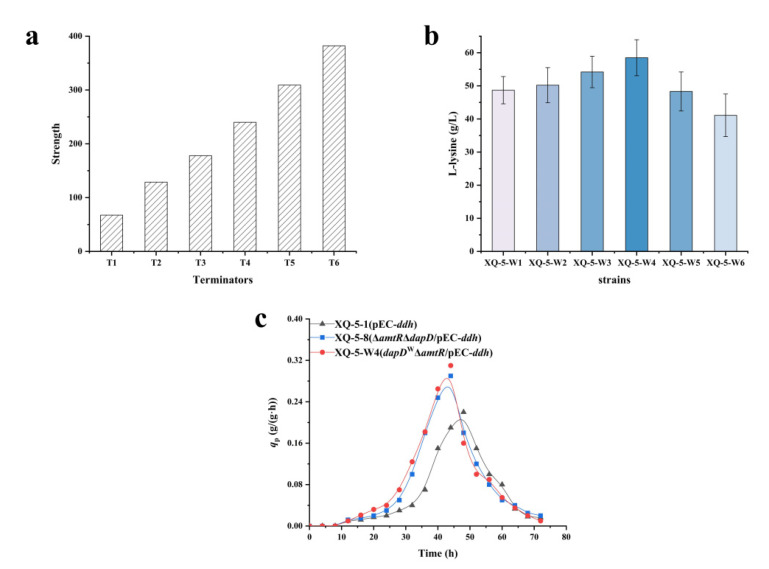
Balancing the flux in the two variants of DAP pathway to promote L-lysine production. (**a**) Different strengths of six terminators. (**b**) L-lysine production of strain XQ-5-W1, XQ-5-W2, XQ-5-W3, XQ-5-W4, XQ-5-W5, and XQ-5-W6 in shake flask culture. (**c**) *q*_Lys_ of strain XQ-5-1, XQ-5-8, and XQ-5-W4 at 300 mM NH_4_^+^ concentration. All data represent values of three determinations of triplicate independent experiments.

There are many ways to weaken genes, including the ones from the level of transcription or translation [37]. At the translation level, weakening the gene is often achieved by replacing RBS to change the gene expression level [38]. In this experiment, terminators were inserted in front of the gene to weaken its expression as the terminators are essential elements controlling the normal transcription of genes [37]. Six terminators with different intensities were selected (Figure 6a) and inserted in front of the *dapD* gene by secondary homologous recombination using pK18*mobSacB* [39]. The sequences of the six terminators are listed in Supplementary material (Table S1). Six recombinant bacteria with different weakening degrees were derived from strain XQ-5-3, i.e., XQ-5-W1, XQ-5-W2, XQ-5-W3, XQ-5-W4, XQ-5-W5, and XQ-5-W6. The L-lysine production of strain XQ-5-W1 (i.e., 48.7 ± 4.12 g/L), XQ-5-W2 (i.e., 50.2 ± 5.31 g/L), XQ-5-W5 (i.e., 48.3 ± 5.87 g/L), and XQ-5-W6 (i.e., 41.1 ± 6.44 g/L) were lower than that of the strain XQ-5-3 (i.e., 53.8 ± 3.98 g/L). Conversely, the L-lysine production of strain XQ-5-W3 (i.e., 54.2 ± 4.76 g/L) and XQ-5-W4 (i.e., 58.5 ± 5.43 g/L) were higher than that of strain XQ-5-3 (i.e., 53.8 ± 3.98 g/L), especially strain XQ-5-W4 (Figure 6b). These results indicated that the introduction of terminators in front of the *dapD* gene could change the translation level of *dapD*, thus affecting the flux in the succinylase pathway. The similar results were also found in previous reports [40]. The highest L-lysine yield was found in strain XQ-5-W4 (i.e., 58.5 ± 5.43 g/L), which was 21.4% higher than that of the original strain XQ-5 (i.e., 48.2 ± 3.54 g/L). In addition, the *q*_Lys,max_ of strain XQ-5-W4 (i.e., 0.31 ± 0.04 g/(g·h)) and strain XQ-5-8 (i.e., 0.30 ± 0.04 g/(g·h)) were similar, about 55% higher than that of strain XQ-5 (i.e., 0.20 ± 0.01 g/(g·h)) (Figure 6c). As expected, the activity of DapD decreased with the increase of terminator strength (i.e., 5.8 ± 0.13 mU/mg-1.3 ± 0.42 mU/mg) (Table 2). In addition, the forward reaction of DapDH increased with the weakening of the succinylase pathway and the reverse reaction was also enhanced (Table 2). It is worth noting that the best balance in strain XQ-5-W4 (i.e., XQ-5-*dapD*^W^Δ*amtR*/pEC-*ddh*) resulted in the best L-lysine yield (i.e., 58.5 ± 5.43 g/L) and *q*_Lys,max_ (i.e., 0.31 ± 0.04 g/(g·h)). At the same time, the by-products of the six strains were measured. In comparison, strain XQ-5-W4 has fewer by-products (Table 3).

### 2.6. Fed-Batch Fermentation of C. glutamicum XQ-5-W4

**Figure 7:**
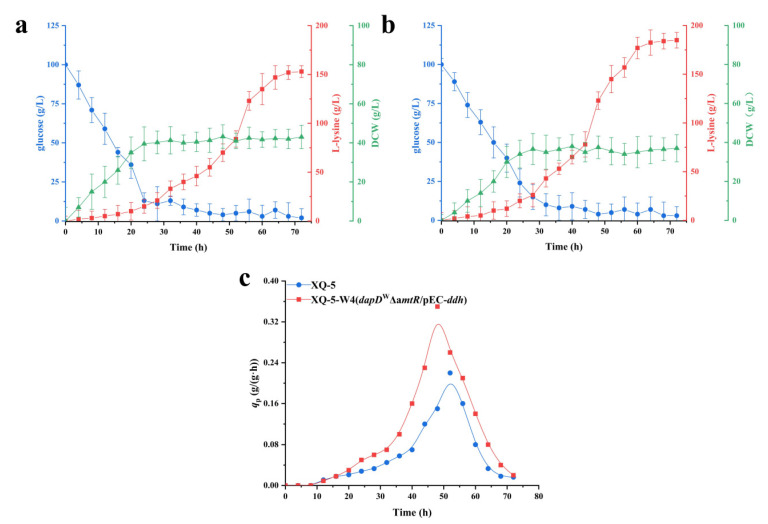
Time course of L-lysine fed-batch fermentations of strains XQ-5 (**a**) and XQ-5-W4 (**b**) in 5-L fermentors. The L-lysine production (squares, red line), DCW (triangle, green line), and glucose (circle, blue line) of strains cultivated in 5-L fermentors. (**c**) *q*_Lys_ of strain XQ-5 and XQ-5-W4 at 300 mM NH_4_^+^ concentration in fed-batch fermentations. All data represent values of three determinations of triplicate independent experiment.

The production performance of strain XQ-5-W4 was investigated in a fed-batch process. As a comparison, fed-batch fermentation of the original strain XQ-5 was also conducted. Figure 7 shows the time profiles of fed-batch fermentations in a 5-L jar fermenter. Fed-batch fermentation of XQ-5-W4 resulted in 189 ± 8.7 g/L of L-lysine with a *q*_Lys,max_ of 0.35 ± 0.05 g/(g·h) (Figure 7b,c). However, fed-batch fermentation of XQ-5 resulted in 151 ± 9.3 g/L of L-lysine with a *q*_Lys,max_ of 0.22 ± 0.02 g/(g·h) (Figure 7a,c). Consistent with the results of production intensity of L-lysine in shake flasks, the *q*_Lys,max_ of strain XQ-5-W4 was higher than that of strain XQ-5 (0.35 ± 0.05 g/(g·h) vs. 0.22 ± 0.02 g/(g·h)) (Figure 7c). In addition, the L-lysine yield of strain XQ-5-W4 stabilized faster, about six hours earlier than that of strain XQ-5. However, the dry weight of strain XQ-5-W4 was 38.9 ± 5.12 g/L, 13.9% lower than that of the original strain (i.e., 45.2 ± 7.64 g/L) (Figure 7a,b). Previous research also found the similar result in which the biomass decreased while enhancing the yield of the target products [23]. Taken together, these results demonstrated that the final strain XQ-5-W4 shows an efficient L-lysine production under fed-batch fermentation, making it a very promising platform for L-lysine production.

## 3. Materials and Methods

### 3.1. Strains, Growth Medium, and Culture Conditions

L-lysine producing strain *C. glutamicum* XQ-5 derived from the wild-type strain *C. glutamicum* ATCC 13032 after multiple rounds of random mutagenesis. Strains and plasmids used in this study are listed in Table 4. Oligonucleotides used in this study are listed in Supplementary Material (Table S2). The L-lysine high-producing strain *C. glutamicum* XQ-5 was derived from the wild-type strain *C. glutamicum* 13032 [41]. Molecular reagents (isopropyl B-D-1-thiogalactopyranoside (IPTG), 2 × Phanta Max Master Mix, 2 × Tag Max Master Mix (Dye Plus) and kanamycin) were purchased from Vazyme Biotech Co., Ltd. (Nanjing, China). Restriction endonucleases and the DNA Ligase were purchased from Thermo Fisher Scientific Shanghai Instruments Co. Ltd. (shanghai, China). Other chemical reagents, including yeast extract, tryptone, and NaCl, were purchased from China National Pharmaceutical Group Corporation (Shanghai, China). The cell concentration was measured with a spectrophotometer (721N, shanghai, China). The glucose and L-lysine concentrations were measured with an SBA-40E immobilized enzyme biosensor (Shandong, China).

*E. coli* grew in Luria-Bertani (LB) medium at 37 °C. *C. glutamicum* grew in LB-glucose (LBG) medium at 30 °C [42]. EPO medium and LB-Brain Heart Infusion-Sorbitol (LBHIS) medium were used to construct the recombinant bacteria [43]. In addition, a 50 μg/mL kanamycin solution was used to build the plasmids and a 25 μg/mL kanamycin solution was used to screen the recombinant strains. A 1 mmol/L IPTG solution was used to induce gene overexpression. Samples were taken from the shake flasks or fermenters every four hours.

The single colony was inoculated in LBG liquid medium and incubated at 30 °C for 12 h with rotation speed 100 r/min. Next, 5 mL of the seed culture was transferred to 50 mL of the fermentation medium in a standard 500 mL shake flask and was cultured for 72 h at 30 °C with rotation speed 100 r/min. The fermentation medium contained (per liter) 100 g glucose, 8 g corn steep liquor, 40 g (NH_4_)_2_SO_4_ (≈300 mM), 0.02 g Fe^+^, 0.02 g Mn^+^, 450 μg VB_1_, 8 mg VB_3_, 850 μg VH, 0.6 mg Zn^+^, 0.53 g KCl, 1 g KH_2_PO_4_, 1 g K_2_HPO_4,_ 4 g MgSO_4_·7H_2_O, 50 mg betaine, 8 mL beet molasses, and 40 g CaCO_3_. Both media were adjusted to pH 7.3 with NaOH. Fermentation conditions: initial pH 7.3 and 10% of inoculation volume.

Fed-batch fermentation was carried out in a 5-L jar fermenter (BLBio-5GJ-2-H, Bailun Bi-Technology Co. Ltd., Shanghai, China). The fermentation medium contained (per liter): 70 g glucose, 20 g corn steep liquor, 2 g KH_2_SO_4_, 50 g beet molasses, 40 g (NH_4_)_2_SO_4_, 1.5 g MgSO_4_.7H_2_0, 0.03 g FeSO_4_, 0.02 g MnSO_4_, 0.03 g glycine betaine, 600 ug biotin, 300 ug thiamine-HCI, and 2 mL antifoam. Ammonia was used to control pH 7.0 and provide nitrogen source for bacteria. The relative dissolved oxygen was controlled at 20–30% by stirring speed and ventilation. The temperature is maintained at 30 °C by jacket cooling. OD_600_, residual sugar concentration and L-Lysine concentration were determined every 4 h during fermentation. The prepared feed solution [44] was used to control the glucose concentration at about 5 g/L by adjusting the feeding rate. L-lysine concentration was determined as lysine·HCl in duplicates.

### 3.2. Analytical Methods

The cell concentration after 25-fold dilution was measured at OD_600_ using a spectrophotometer. The correlation coefficient between the dry cell weight (DCW) and OD_600_ was 0.32 (1 OD_600_ = 0.32g DCW). After sample dilution of 100 times, glucose and L-lysine concentrations were measured with an SBA-40E immobilized enzyme biosensor. The concentration of the by-products was measured with high performance liquid chromatography (HPLC) [44]. Cell morphology was observed via field emission scanning electron microscopy (FESEM). Cells of *C. glutamicum* in the mid-log phase were collected by centrifugation and rinsed three times in physiological saline (pH 7.0). Bacterial cells were spread onto a small silicon platelet and air dried under room temperature, followed by in-situ fixation with a 2.5% glutaraldehyde solution in a 0.15 M sodium phosphate buffer (pH7.4) for 10 min. The samples were coated with gold and transferred to FESEM (SU8220, Hitachi, Japan) for observation at an accelerating voltage of 3 kV.

### 3.3. Construction of C. glutamicum Recombinant Strains

Restriction endonucleases and the DNA Ligase were used to construct the plasmids. In this study, the plasmid pEC-XK99E was used for gene overexpression in *C. glutamicum*. The suicide plasmid pK18*mobsacB* was used for gene knockout in *C. glutamicum.* Firstly, the constructed plasmid was electroporated into *C. glutamicum*, and then the positive transformants were screened with a 25 μg/mL kanamycin solution in LBH medium. The final positive transformants were obtained by eliminating the plasmids according to the sucrose lethal principle. The deletions in the chromosome were verified by PCR analysis.

### 3.4. Real-Time PCR

In order to analyze RNA, cells in exponential phase were collected during shake flask fermentation for mRNA isolation. RNA was extracted with an RNAiso Plus reagent (Takara, Dalian, China). The cDNA was synthesized with RevertAid^TM^ First Strand cDNA synthesis kit (Fermentas, Shanghai, China). The Ct values of the 16S rDNA gene and those of the *ddh* and *dapD* genes were obtained by RT-qPCR using a Bio-Rad CFX96 Touch Real-Time PCR Detection System (Bio-Rad Hercules, CA, USA) with SYBR Premix Ex Taq^TM^ II (Takara, Dalian, China). The primer sequences used for RT-qPCR and RT-PCR are shown in Table S3. Each sample was analyzed in triplicate.

### 3.5. Preparation of Crude Extracts and Enzyme Assays

Crude enzyme solution was prepared to measure the activities of DapDH and DAPD. The preparation method was based on a previous report [45]. Enzyme activity was analyzed in triplicate.

DapDH activity was measured at 30 °C. The forward reaction mixture contained 200 mM glycine-KOH (pH 10.5), 100 mM (NH_4_)_2_SO_4_, 0.3 mM NADPH, 5 mM THDPA, and crude enzymes extract. The reverse reaction mixture contained 200 mM glycine/NaOH (pH 10.5), 2 mM NADP^+^, 4 mM *meso*-DAP, and crude enzymes extract. One unit is defined as the amount of enzyme which catalyzes the formation or decrease of 1 μmol NADPH (340 nm) per minute [46].

DapD activity was measured by the formation of free coenzyme A (CoA) at 412 nm. The reaction mixture contained 0.1 M Tris-HCI (pH 8.0), 0.5 mM DTNB, 0.2 mM succinyl-CoA, 5 mM 2-aminopimelate, and crude enzymes extract. One unit is defined as the amount of enzyme which catalyzes the formation of 1 μmol CoA per minute [32].

## 4. Conclusions

For the first time, DAP pathway was reconstructed to optimize L-lysine production in *C. glutamicum*, which demonstrated that the dehydrogenase pathway is promising for promoting L-lysine production. In *C. glutamicum*, both the dehydrogenase pathway and the succinylase pathway are involved in the production of L-lysine, but the relative proportion of each pathway on L-lysine biosynthesis is different because of the different demand for NH_4_^+^ concentration [16]. The proportion of the dehydrogenase pathway on L-lysine production increased when increasing the NH_4_^+^ concentration (Figure 2d). Since the L-lysine biosynthesis in the dehydrogenase pathway has less steps, the strain showed the highest *q*_Lys,max_ at high NH_4_^+^ concentration (Figure 2c). The similar results were also found in the AmtR-deficient strain (Figure 4b), as NH_4_^+^ was efficiently transferred into the cell during inactivation of AmtR [25]. The L-lysine yield and *q*_Lys,max_ in strain XQ-5-2 reached 52.3 ± 4.31 g/L and 0.22 ± 0.03 g/(g·h), which were 8.5% and 10% higher than that of the original strain XQ-5, respectively. Although the dehydrogenase pathway is promising in promoting L-lysine production, redirecting the flux into the dehydrogenase pathway while blocking the succinylase pathway is counterproductive to L-lysine production (Figure 5d). Fortunately, this problem can be overcome by weakening the succinylase pathway (Figure 6b). The target strain *C. glutamicum* XQ-5-W4 produced 58.5 ± 5.43 g/L L-lysine with a *q*_Lys,max._ (i.e., 0.31 ± 0.04 g/(g·h)) in shake-flask fermentation, which were 21.4% and 55% higher than that of strain XQ-5. In addition, fed-batch fermentation of strain XQ-5-W4 resulted in 189 ± 8.7 g/L of L-lysine with a *q*_Lys,max_ of 0.35 ± 0.05 g/(g·h). These results indicated that the reconstruction of DAP pathway to switch the flux in the variants of DAP pathway has great potential to promote L-lysine production in *C. glutamicum*.

## Figures and Tables

**Table 1:** By-products of *Corynebacterium glutamicum* strains XQ-5-1, XQ-5-3, and XQ-5-8.

	XQ-5-1 (XQ-5/pEC-*ddh*)			XQ-5-3 (XQ-5-Δ*amtR*/pEC-*ddh*)			XQ-5-8 (XQ-5-Δ*amtR*Δ*dapD*/pEC-*ddh*)		
by-Products	200 mM	300 mM	400 mM	200 mM	300 mM	400 mM	200 mM	300 mM	400 mM
glutamate	0.26 ± 0.05	0.35 ± 0.11	0.54 ± 0.02	0.49 ± 0.06	0.97 ± 0.11	1.23 ± 0.02	1.07 ± 0.04	1.75 ± 0.15	2.01 ± 0.02
pyruvate	1.94 ± 0.04	2.08 ± 0.14	2.36 ± 0.02	2.21 ± 0.04	2.65 ± 0.03	3.32 ± 0.04	3.01 ± 0.12	3.43 ± 0.09	3.91 ± 0.12
isoleucine	1.06 ± 0.16	1.33 ± 0.11	1.35 ± 0.12	1.57 ± 0.02	2.03 ± 0.03	2.37 ± 0.02	2.31 ± 0.04	2.93 ± 0.11	3.51 ± 0.02
aspartate	2.06 ± 0.06	2.87 ± 0.05	2.92 ± 0.07	2.98 ± 0.08	3.52 ± 0.12	3.91 ± 0.12	3.88 ± 0.16	4.01 ± 0.11	4.22 ± 0.12
methionine	1.12 ± 0.11	1.33 ± 0.07	1.41 ± 0.15	1.22 ± 0.02	1.54 ± 0.11	1.87 ± 0.08	1.46 ± 0.02	1.98 ± 0.05	2.31 ± 0.06
threonine	0.98 ± 0.13	1.15 ± 0.14	1.23 ± 0.02	1.06 ± 0.16	2.01 ± 0.11	3.91 ± 0.12	2.17 ± 0.13	3.23 ± 0.11	3.91 ± 0.12

All data represent values of three determinations of triplicate independent experiments.

**Table 2:** The activity DapDH and DapD different recombinant *C. glutamicum* strains.

Strains	Specific Activity (mU/mg of Protein)		
	DapD	DapDH(F-Reaction)	DapDH (R-Reaction)
XQ-5-W1	5.8 ± 0.13	201 ± 13.6	130 ± 13.8
XQ-5-W2	5.3 ± 0.21	227 ± 22.1	138 ± 18.1
XQ-5-W3	4.5 ± 0.39	241 ± 26.8	145 ± 17.4
XQ-5-W4	3.3 ± 0.26	260 ± 18.4	151 ± 21.2
XQ-5-W5	2.1 ± 0.22	268 ± 19.3	177 ± 19.3
XQ-5-W6	1.3 ± 0.42	275 ± 20.2	194 ± 18.9

All data represent values of three determinations of triplicate independent experiments.

**Table 3:** By products of *C. glutamicum* strains XQ-5-W1, XQ-5-W2, XQ-5-W3, XQ-5-W4, XQ-5-W5, and XQ-5-W6.

Strains	by-Products (g/L)					
	Glutamate	Pyruvate	Isoleucine	Aspartate	Methionine	Threonine
XQ-5-W1	0.4 ± 0.03	2.11 ± 0.14	1.5 ± 0.09	2.93 ± 0.18	1.54 ± 0.04	1.26 ± 0.08
XQ-5-W2	0.54 ± 0.04	2.23 ± 0.13	1.64 ± 0.07	2.85 ± 0.12	1.25 ± 0.02	1.75 ± 0.11
XQ-5-W3	0.64 ± 0.12	2.41 ± 0.10	1.32 ± 0.05	2.42 ± 0.12	1.05 ± 0.06	1.35 ± 0.02
XQ-5-W4	0.78 ± 0.04	2.32 ± 0.13	1.24 ± 0.09	2.15 ± 0.11	0.85 ± 0.12	1.65 ± 0.08
XQ-5-W5	1.32 ± 0.07	2.87 ± 0.11	2.01 ± 0.12	2.78 ± 0.05	1.05 ± 0.10	2.75 ± 0.02
XQ-5-W6	1.64 ± 0.12	3.12 ± 0.03	2.64 ± 0.14	3.65 ± 0.02	1.75 ± 0.04	3.01 ± 0.12

All data represent values of three determinations of triplicate independent experiments.

**Table 4:** The main strains and plasmids used in this study.

Strains and Plasmids	Characters	Reference
*C. glutamicum* strains		
XQ-5	*C. glutamicum* AEC^r^ 2-TA^r^ MF^r^ Met^s^, L-lysine-producing bacteria derived from strain *C. glutamicum* ATCC13032	[41]
XQ-5-1	strain XQ-5 harboring plasmid pEC-XK99E-*ddh*	this study
XQ-5-2	derivative of strain XQ-5 with deletion of *amtR*	this study
XQ-5-3	strain XQ-5-2 harboring plasmid pEC-XK99E-*ddh*	this study
XQ-5-4	derivative of strain XQ-5 with deletion of *dapD*	this study
XQ-5-5	derivative of strain XQ-5 with deletion of *ddh*	this study
XQ-5-6	derivative of strain XQ-5-2 with deletion of *dapD*	this study
XQ-5-7	strain XQ-5-4 harboring plasmid pEC-XK99E-*ddh*	this study
XQ-5-8	strain XQ-5-6 harboring plasmid pEC-XK99E-*ddh*	this study
XQ-5-W1	derivative of strain XQ-5-3 with weaking of *dapD*(T1-Terminator)	this study
XQ-5-W2	derivative of strain XQ-5-3 with weaking of *dapD*(T2-Terminator)	this study
XQ-5-W3	derivative of strain XQ-5-3 with weaking of *dapD*(T3-Terminator)	this study
XQ-5-W4	derivative of strain XQ-5-3 with weaking of *dapD*(T4-Terminator)	this study
XQ-5-W5	derivative of strain XQ-5-3 with weaking of *dapD*(T5-Terminator)	this study
XQ-5-W6	derivative of strain XQ-5-3 with weaking of *dapD*(T6-Terminator)	this study
plasmid		
pEC-XK99E	Kan^r^, Expression vector with *pMB1* replicon	stratagene
pK18mobSacB	Kan^r^, Integration vector	stratagene
pEC-XK99E-*ddh*	pEC-XK99E carrying gene *ddh* from *C.glutamicum*	this study
pK18*mobsacB*-Δ*amtR*	pK18*mobsacB* carrying *amtR*-L and *amtR*-R fragments	this study
pK18*mobsacB*-Δ*dapD* pK18*mobsacB*-Δ*ddh*	pK18*mobsacB* carrying *dapD*-L and *dapD*-R fragments	this study
	pK18*mobsacB* carrying *ddh*-L and *ddh*-R fragments	this study
pK18*mobsacB*-T1	a derivative of pK18*mobsacB*, harboring the fragment of inserting T1 terminator in front of *dapD*	this study
pK1*8mobsacB*-T2	a derivative of pK18*mobsacB*, harboring the fragment of inserting T2 terminator in front of *dapD*	this study
pK18*mobsacB*-T3	a derivative of pK18*mobsacB*, harboring the fragment of inserting T3 terminator in front of *dapD*	this study
pK18*mobsacB*-T4	a derivative of pK18*mobsacB*, harboring the fragment of inserting T4 terminator in front of *dapD*	this study
pK18*mobsacB*-T5	a derivative of pK18*mobsacB*, harboring the fragment of inserting T5 terminator in front of *dapD*	this study
pK18*mobsacB*-T6	a derivative of pK18*mobsacB*, harboring the fragment of inserting T6 terminator in front of *dapD*	this study
XQ-5-W4	derivative of strain XQ-5-3 with weaking of *dapD*(T4-Terminator)	this study
XQ-5-W5	derivative of strain XQ-5-3 with weaking of *dapD*(T5-Terminator)	this study
XQ-5-W6	derivative of strain XQ-5-3 with weaking of *dapD*(T6-Terminator)	this study

## Data Availability

The data presented in this study are available on request from the corresponding author.

## Supplementary Materials

The following are available online at https://www.mdpi.com/article/10.3390/ijms22169065/s1.
