# Supplementary material for: Reconstruction of the Diaminopimelic Acid Pathway to Promote L-lysine Production in Corynebacterium glutamicum
Source: Int J Mol Sci. 2021 Aug 23;22(16):9065. doi: 10.3390/ijms22169065 (PMC8396482; doi:10.3390/ijms22169065)
Supplement: Supplementary file 1 [file ijms-22-09065-s001.zip › ijms-1338671-SI.pdf]

Reconstruction of the diaminopimelic acid pathway to promote L-lysine production in *Corynebacterium glutamicum*

Ning Liu[1], Ting-Ting Zhang[1], Zhi-Ming Rao[1,2,*], Wei-Guo Zhang[1], Jian-Zhong Xu[1,*]

[1] The Key Laboratory of Industrial Biotechnology, Ministry of Education, School of Biotechnology, Jiangnan University, 1800# Lihu Road, WuXi 214122, People's Republic of China;6190206010@stu.jiangnan.edu.cn (N.L.); ln576678072@163.com (T.-T.Z.); zhangwg@jiangnan.edu.cn (W.-G.Z.)

[2] National Engineering Laboratory for Cereal Fermentation Technology (NELCF), Jiangnan University, 1800# Lihu Road, Wuxi, 214122, People's Republic of China

[*] Correspondence: xujianzhong@jiangnan.edu.cn (J.-Z.X.); raozhm@jiangnan.edu.cn (Z.-M.R.);

Tel./Fax: +86-510-85329312 (J.-Z.X.)

The build processes of recombinant plasmids

The plasmid pEC-XK99E-*ddh* construction

The nucleotide sequences of the gene *ddh* from *C. glutamicum* XQ-5 with two restriction sites (i.e., *EcoR*I and *Xba*I) were amplified by PCR, and then ligated with plasmid pEC-XK99E. The resulted plasmid was designated as pEC-XK99E-*ddh*.

The plasmid pK18*mobsacB*-Δ*amtR* construction

The homology arm sequences of the gene *amtR* from *C. glutamicum* XQ-5 with two restriction sites (i.e., *EcoR*I and *Xba*I) were amplified by fusion PCR, and then ligated with plasmid pK18*mobsacB*. The resulted plasmid was designated as pK18*mobsacB*-Δ*amtR.*

The plasmid pK18*mobsacB*-Δ*dapD* construction

The homology arm sequences of the gene *dapD* from *C. glutamicum* XQ-5 with two restriction sites (i.e., *Sma*I and *Hin*dIII) were amplified by fusion PCR, and then ligated with plasmid pK18*mobsacB*. The resulted plasmid was designated as pK18*mobsacB*-Δ*dapD.*

The plasmid pK18*mobsacB*-Δ*ddh* construction

The homology arm sequences of the gene *ddh* from *C. glutamicum* XQ-5 with two restriction sites (i.e., *EcoR*I and *Xba*I) were amplified by fusion PCR, and then ligated with plasmid pK18*mobsacB*. The resulted plasmid was designated as pK18*mobsacB*-Δ*ddh.*

The plasmid pK18*mobsacB*-T1, T2, T3, T4, T5, T6 construction

The T1 terminator and the left homologous arm of gene *dapD* were designed on the same primer, and then the gene *dapD* homologous arm sequences were amplified by fusion PCR, and then ligated

with plasmid pK18*mobsacB*. The resulted plasmid was designated as pK18*mobsacB*-T1. Construction of plasmid pK18mobsacB- T2, T3, T4, T5, T6 were similar to that of plasmid pK18*mobsacB*-T1.

The procedures of recombinant strain constructions

Construction of XQ-5-2 (Δ*amtR*), XQ-5-4 (Δ*dapD*), XQ-5-5 (Δ*ddh*) and XQ-5-6 (Δ*amtR*Δ*dapD*)

The plasmid pK18*mobsacB*-Δ*amtR*, pK18*mobsacB*-Δ*ddh and* pK18*mobsacB*-Δ*dapD* were electroporated into *C. glutamicum* XQ-5 , and then the positive transformants were screened with 25μg /mL concentration of kanamycin in LBH medium. The final positive transformants were obtained by eliminating the plasmids according to the sucrose lethal principle. The deletion of genes *amtR*, *ddh* and *dapD* were analyzed by PCR, and strain XQ-5-2, XQ-5-4 and XQ-5-5 were obtained. The strain XQ-5-6 was based on strain XQ-5-2 by deleting the gene dapD through the same steps.

Construction of XQ-5-1 (XQ-5/pEC-*ddh*), XQ-5-3 (Δ*amtR*/pEC-*ddh*), XQ-5-7 (Δ*dapD*/pEC-*ddh*) and XQ-5-8 (Δ*amtR*Δ*dapD* /pEC-*ddh*)

The plasmid pEC-XK99E-*ddh* was transformed into XQ-5, XQ-5-2, XQ-5-4 and XQ-5-6, respectively, and then resulting in XQ-5-1, XQ-5-3, XQ-5-7 and XQ-5-8, respectively.

Construction of XQ-5-W1, XQ-5-W2, XQ-5-W3, XQ-5-W4, XQ-5-W5 and XQ-5-W6.

The plasmid pK18*mobsacB*-T1, pK18*mobsacB*-T2, pK18*mobsacB*-T3, pK18*mobsacB*-T4, pK18*mobsacB*-T5 and pK18*mobsacB*-T6 were electroporated into strain XQ-5-3 (Δ*amtR*/pEC-*ddh*), and then the positive transformants were screened with 25μg /mL concentration of kanamycin in LBH medium. The final positive transformants were obtained by eliminating the plasmids according to the sucrose lethal principle. The strain XQ-5-W1, XQ-5-W2, XQ-5-W3, XQ-5-W4, XQ-5-W5 and XQ-5-W6 were obtained by PCR analysis.

Table S1 The Sequence of Terminators

name	terminator sequence (5'-3')	strength
T1	CCAATTATTGAAGCGGCTAACGCCGCTTTTTTTGTTTCTGGT	67.43
T2	CCAATTATTGAACACCCTAACGGGTGTTTTTTTGTTTCTGGT	128.86
T3	GACGAACAATAAGGCCTCCCTAACGGGGGGCCTTTTTTATT	177.94
T4	CTCGGTACCAAATTCCAGAAAAGAGACGCTGAAAAGCGTC	239.91
T5	CTCGGTACCAAAAAAAAAAAAAAAAGACACTGAAAAGCGT	309.20
T6	GGAAACACAGAAAAAAAGCCCGCACCTGACAGTGCGGGCT	382.13

Table S2 The oligonucleotides used in this study

**primer**	**sequence (5’-3’)**	restriction sites
amtR-L-F	CCGGAATTCGAATCACTCGTGCTTCACCCC	*EcoR*I
amtR-L-R	TTGGCCTATTGATCTCAAGAGATTGTAGCCAGTA	
amtR-R-F	AGATCAATAGGCCAACTCGCCACCGAAATCGT	
amtR-R-R	TGCTCTAGAGTGCAGCCTGGTGGATCCATT	*Xba*I
dapD-L-F	TCCCCCGGGGAACCGTGGATTCCTTCGCT	*Sma*I
dapD -L-R	GAATAATTTCTATCGGCCTATCAGTTCAAGT	
dapD -R-F	TAGAAATTATTCCCTAGCCTGGATCCGATC	
dapD -R-R	CCCAAGCTTCTACCTCGCCTCCATCATGAT	*Hind*III
ddh-L-F	CCGGAATTCTCACGGATGCTGTTGGGCA	*EcoR*I
ddh-L-R	TTGTAATCCTCCAAAATTGTGGTGGCACTGTC	
ddh-R-F	TTTGGAGGATTACAATCGAGGGGCAAGGAAACA	
ddh-R-R	TGCTCTAGACGTCACAAACCCATCTTTAG	*Xba*I
pEC-*ddh*-F	CCGGAATTCCGTTCGCTTTCGGTCCTGAT	*EcoR*I
pEC-*ddh*-R	CTAGTCTAGAGTCACGCCTGTCTGGTTTCCT	*Xba*I
dapD^{W1}-L-F	CCGGAATTCCGCTTTCCTCATCATTGGT	*EcoR*I
dapD^{W1}-L-R	ACCAGAAACAAAAAAAGCGGCGTTAGCCGCTTC<br>AATAATTGGCCTATCAGTTCAAGTCGGAAGG	
dapD^{W1}-R-F	AGCGGCTAACGCCGCTTTTTTTGTTTCTGGTCCG<br>ATAGAAATTATTCTGGACGTC	
dapD^{W1}-R-R	CCCAAGCCTGTCCGTTGGTGAAAGCGAT	*Hind*III
dapD^{W2}-L-F	CCGGAATTCCGCTTTCCTCATCATTGGT	*EcoR*I
dapD^{W2}-L-R	CCAGAAACAAAAAAACACCCGTTAGGGTGTTCA<br>ATAATTGGCCTATCAGTTCAAGTCGGAAGG	
dapD^{W2}-R-F	ACACCCTAACGGGTGTTTTTTTGTTTCTGGTCCGA<br>TAGAAATTATTCTGGACGTC	
dapD^{W2}-R-R	CCCAAGCCTGTCCGTTGGTGAAAGCGAT	*Hind*III
dapD^{W3}-L-F	CCGGAATTCCGCTTTCCTCATCATTGGT	*EcoR*I
dapD^{W3}-L-R	AATAAAAAAGGCCCCCCGTTAGGGAGGCCTTATTG<br>TTCGTCCCTATCAGTTCAAGTCGGAAGG	
dapD^{W3}-R-F	AGGCCTCCCTAACGGGGGGCCTTTTTTATTCCGATA<br>GAAATTATTCTGGACGTC	
dapD^{W3}-R-R	CCCAAGCCTGTCCGTTGGTGAAAGCGAT	*Hind*III
dapD^{W4}-L-F	CCGGAATTCCGCTTTCCTCATCATTGGT	*EcoR*I
dapD^{W4}-L-R	GACGCTTTTCAGCGTCTCTTTTCTGGAATTTGGTAC<br>CGAGCCTATCAGTTCAAGTCGGAAGG	
dapD^{W4}-R-F	AATTCCAGAAAAGAGACGCTGAAAAGCGTCCCGA<br>TAGAAATTATTCTGGACGTC	
dapD^{W4}-R-R	CCCAAGCCTGTCCGTTGGTGAAAGCGAT	*Hind*III
dapD^{W5}-L-F	CCGGAATTCCGCTTTCCTCATCATTGGT	*EcoR*I
dapD^{W5}-L-R	ACGCTTTTCAGTGTCTTTTTTTTTTTTTTTGGTACCG<br>AGCCTATCAGTTCAAGTCGGAAGG	

Refer to Table S2 (continued)

**primer**	**sequence (5'-3')**	restriction sites
dapD^{W5}-R-F	AAAAAAAAAAAAAAAAGACACTGAAAAGCGTCCGA TAGAAATTATTCTGGACGTC	
dapD^{W5}-R-R	CCCAAGCCTGTCCGTTGGTGAAAGCGAT	*Hind*III
dapD^{W6}-L-F	CCGGAATTCCGCTTTCCTCATCATTGGT	*EcoR*I
dapD^{W6}-L-R	GCCCGCACTGTCAGGTGCGGGCTTTTTTCTGTGTTT CCCCTATCAGTTCAAGTCGGAAG	
dapD^{W6}-R-F	GAAAAAAGCCCGCACCTGACAGTGCGGGCTCCGAT AGAAATTATTCTGGACGTC	
dapD^{W6}-R-R	CCCAAGCCTGTCCGTTGGTGAAAGCGAT	*Hind*III

Table S3 Primers used for RT-PCR in this work

primers	sequence (5'-3')
16SRNA-F:	ACCTGGAGAAGAAGCACCG
16SRNA-R	TCAAGTTATGCCCGTATCG
ddh-F	CGTGCTGTTCCTGTGCATG
ddh-R	AGAACATTCCTGGATCCCAGC
dapD-F	GCCTCCTCAACAATGTCGTG
dapD-R	CACGAAGCCCTCATGCATCA
